# Supplementary material for: SARS-CoV-2 induces robust germinal center CD4 T follicular helper cell responses in rhesus macaques
Source: Nat Commun. 2021 Jan 22;12:541. doi: 10.1038/s41467-020-20642-x (PMC7822826; doi:10.1038/s41467-020-20642-x)
Supplement: Supplementary file 1 — Supplementary Information [file 41467_2020_20642_MOESM1_ESM.pdf]

SUPPLEMENTARY FIGURE TITLES AND LEGENDS

Supplementary Table 1. Antibody and chemical information. All antibodies and chemicals used in the study are denoted.

Supplementary Table 2. Animal information. Sex, Age, Body weight, treatment, infusion volume, blood donor type, total volume infused, necropsy date, prior treatments and extra notes for all animals on the study.

Supplementary Table 3a. Spleen AIM Assay conditions. N, S, M, ORF (nsp3, nsp4, 3a, 8), or P/I stimulation conditions for each animal on the study.

Supplementary Table 3b. Mediastinal LN AIM Assay conditions. N or S peptide stimulation conditions for mediastinal LN for each animal on the study.

1. Supplementary Table 1

ANTIBODIES

Reagents	Clone	Source	Catalog number	Lot Number	Concentration for staining
CD3-AF 700	SP34-2	BD Biosciences	557917	9277122	0.2 mg/ml
CD3-APC-Cy7	SP34-2	BD Biosciences	557757	9252411	0.2 mg/ml
CD4-BV 650	L200	BD Biosciences	563737	9036946	0.25 mg/ml
CD8-BV 510	SK-1	BD Biosciences	563919	9344072	0.2 mg/ml
CD8-BUV 805	SK-1	BD Biosciences	564913	0195677	0.2 mg/ml
CD20-APC-Cy7	2H7	BioLegend	302314	B288789	0.2 mg/ml
CD20-BV 421	2H7	BioLegend	302328	B285815	0.1 mg/ml
CD95-BUV 737	DX2	BD Biosciences	564710	0192242	0.2 mg/ml
CD279(PD-1)-Pe-Cy7	EH12.2H8	BioLegend	329918	B272021	0.2 mg/ml
CX3CR1-PE-CF594	2A9-1	BioLegend	341624	B295187	0.1 mg/ml
CXCR3-BV 786	IC6	BD Biosciences	741005	0078361	0.05 mg/ml
CXCR5-PE	MU5UBEE	Thermofischer	12-9185-411G1	2119063	0.125 mg/ml
CCR4-BV 605	1G1	BD Biosciences	562906	9290304	0.24 mg/ml
CCR6-PE-CF594/A610	G034E3	BioLegend	353430	B298181	0.05 mg/ml
CCR7-BV 650	3D12	BD Biosciences	563407	0078602	0.2 mg/ml
HLA-DR-BV 786	L243	BioLegend	307642	B283993	0.1 mg/ml

CD69-BV 711	FN50	BioLegend	310944	B277989	0.1 mg/ml
CD69-Pe-Cy7	FN50	Invitrogen	25-0699-42	2165779	0.06 mg/ml
CD14-AF 700	MSE2	BD Biosciences	301822	B285400	0.5 mg/ml
CD16-BV 605	3G8	BD Biosciences	563172	9179026	0.12 mg/ml
CD11b-BV 510	ICRF44	Thermofisher	563088	B244424	0.2 mg/ml
CD11c-Pe-Cy7	3.9	Invitrogen	25-0116-42	2142959	0.2 mg/ml
CD103-APC	2G5	Beckman Coulter	B06204	200042	0.1 mg/ml
CD66-APC	TET2	Miltenyi	130-118-539	5200300765	1:50 dilution
CD163-PE	GHI/61	BioLegend	333606	B289302	0.2 mg/ml
CD123-BV 421	7G3	Thermofisher	501129764	2181846	0.2 mg/ml
Granzyme B-BV 421	GB11	BioLegend	515408	B301155	0.1 mg/ml
CD80-AF 488	2D10.4	Invitrogen	11-0809-42	2144183	0.2 mg/ml
CD86-AF 488	IT2.2	BioLegend	305414	B243405	0.4 mg/ml
Ki-67-AF 488	B56	BD Biosciences	558616	9123835	0.05 mg/ml
Ki-67-BV 510	B56	BD Biosciences	563462	9301839	0.4 mg/ml
CD28-Pe-Cy7	CD28.2	Tonbo	40-0289-U500	B259245	0.05 mg/ml
a4b7-PE	Act-1	NHP Reag Res	PR-1422	EP021219	0.2 mg/ml
CD45-AF 488	D058-1283	BD Biosciences	557803	9311681	0.025 mg/ml
CD45-BV 605	D058-1283	BD Biosciences	564098	9051992	0.2 mg/ml
CD140b-APC	18A2	BioLegend	323608	B285844	0.3 mg/ml
Bcl-6-APC-Cy7	K112-91	BD Biosciences	563581	0050675	0.1 mg/ml
CD21- PE-CF594	B-ly4	BD Biosciences	563474	0066046	0.1 mg/ml
SLAM-AF 488	A12(7D4)	BioLegend	306312	B268886	0.4 mg/ml
Foxp3-APC	206D	BioLegend	320114	B241845	0.03 mg/ml
CD278 (ICOS)-BV 785	C396.4A	BioLegend	313534	305759	0.15 mg/ml
CD25-APC	BC96	Tonbo	20-0259-T100	C0259082 118203	0.25 mg/ml

CD 134 (OX40)-BV 786	L106	BD Biosciences	744746	0170918	0.1 mg/ml
4-1BB-AF 488	4B4-1	BD Biosciences	11-1379-42	281868	0.2 mg/ml
TNFa-AF 488	Mab11	BioLegend	502906	B271495	0.1 mg/ml
IL-21-APC	3A3-N2.1	BD Biosciences	560493	9199272	0.025 mg/ml
IFNG-PeCy7	B27	BioLegend	506518	B255741	0.1 mg/ml
IL2- PE-CF594	MO1-17H12	BioLegend	500344	B245312	0.025 mg/ml
CD107a-PE	H4A3	BioLegend	328608	B283846	0.4 mg/ml
CD107b-PE	EbioH4B4	Thermofischer	12-1078-42		0.1 mg/ml
IL-17-BV 421	eBio64DEC17	eBiosciences	48-7179-42	2181862	0.2 mg/ml
CD45 RO-PE-CF594	UCHL-1	BD Biosciences	562299	9078806	0.2 mg/ml
CD28-Purified Ab	CD28.2	BD Biosciences	555725	9266655	0.5 mg/ml
CD49d-Purified Ab	9F10	BD Biosciences	555501	9154508	0.5 mg/ml
Live/dead-APC-Cy7		Life technologies	L34976	2192281	1:100
Live/dead-BV 510		Life technologies	L34966	1899019	1:100
Bcl-6	LN22	Biocare Medical	CM410A	080420A	1:100
CD3	CD3-12	Abcam	Ab11089	GR336112 5-1	1:100
PD-1	Polyclonal	Novus	NBP1-88104	000000440	1:100

2. Chemicals:

Reagents	Source	Identifier (Cat#)	Lot Number	Concentrations
Golgi Stop	BD Biosciences	554724	9213627-A	3 mM
Golgi Plug	BD Biosciences	555029	9284211	1mg/ml
PMA+ Ionomycin cocktail mix	eBiosciences	00-4970-93	2157128	500X
T cell activation/ expansion kit NHP	MACS Miltenyi Biotech	130-092-919	519083-132	10 ug/antibody/ml 5ul/ test/ one million cells
AIM V media	Gibco	12055091	2166131	
RPMI media-1640	Gibco	11875098	2183709	
Cytofix/cytoperm	BD Biosciences	51-2090K2	6292704	100 ul/test
DNAse-I	Roche diagnostics	BM070	00876819	30 U/ml
Collagenase-type IV	Worthington Biomedical corporation	LS004188	49A19027	250 U/ml

Supplementary Table 2. Animal information

Animal code	Sex	Age	Body Weight (kg)	Treatment	Infusion Volume	Total volume infused (4ml/kg)	Nx Day	Prior treatments	Clinical Notes
Control 1	**F**	5:10:09	**6**	**no plasma**	**N/A**	**N/A**	D11	44470 received clinical analgesics and antibiotics for trauma and nutritional supplements due to lean BCS	
Control 2	**F**	4:10:07	**7.01**	**no plasma**	**N/A**	**N/A**	D11		Sneezed during study
CP1	**M**	5:10:14	**8.43**	**Conval. Plasma**	**27ml**	**33.7**	D12	Dexamethasone suppression/ACTH stimulation tests as part of BBA testing (2014) Experimental vaccine (for Campylobacter coli in 2018)	
NP1	**M**	5:10:26	**8.95**	**normal plasma**	**27ml**	**35.8**	D12	44309 has historically received clinical analgesics, antibiotics, and supplements for trauma cases	
NP2	**M**	5:09:18	**9.74**	**normal plasma**	**30ml**	**39.0**	D13		
CP2	**M**	5:10:19	**8.83**	**Conval. Plasma**	**27ml**	**35.3**	D13	44379 has received analgesics/antibiotics for trauma and antibiotics and probiotics for diarrhea historically	Sneezed during study
Control 3	**F**	4:10:23	**5.47**	**no plasma**	**N/A**	**N/A**	D14	45159 has received analgesics/antibiotics for trauma and probiotics for diarrhea	Dermatitis
Control 4	**M**	4:10:00	**10.72**	**no plasma**	**N/A**	**N/A**	D14	45359 has received analgesics/antibiotics for trauma and probiotics for diarrhea	

Supplementary Table 3a: Spleen AIM Assay conditions

Spleen	Animal code	N	S	M	ORF1-nsp3	ORF1-nsp4	ORF3a	ORF8	P/I
**1**	Control 1	Excluded due to low CD3 events	✓	✓	✓	✓	✓	✓	✓
**2**	Control 2	✓	✓	✓	✓	✓	✓	✓	✓
**3**	CP1	✓	✓	✓	✓	✓	✓	✓	✓
**4**	NP1	✓	✓	✓	✓	✓	✓	✓	✓
**5**	NP2	Excluded due to low recovery of CD95+ cells							
**6**	CP2								
**7**	Control 3	✓	✓	✓	✓	✓	✓	✓	✓
**8**	Control 4	✓	✓	✓	✓	✓	✓	✓	✓

Supplementary Table 3b: Mediastinal LN AIM Assay conditions

Med LN	Animal code	N	S
**1**	Control 1	Excluded due to low recovery of CD95+ cells	
**2**	Control 2		
**3**	CP1	✓	✓
**4**	NP1	✓	✓
**5**	NP2	Excluded due to low recovery of CD95+ cells	
**6**	CP2		
**7**	Control 3	✓	✓
**8**	Control 4	✓	✓

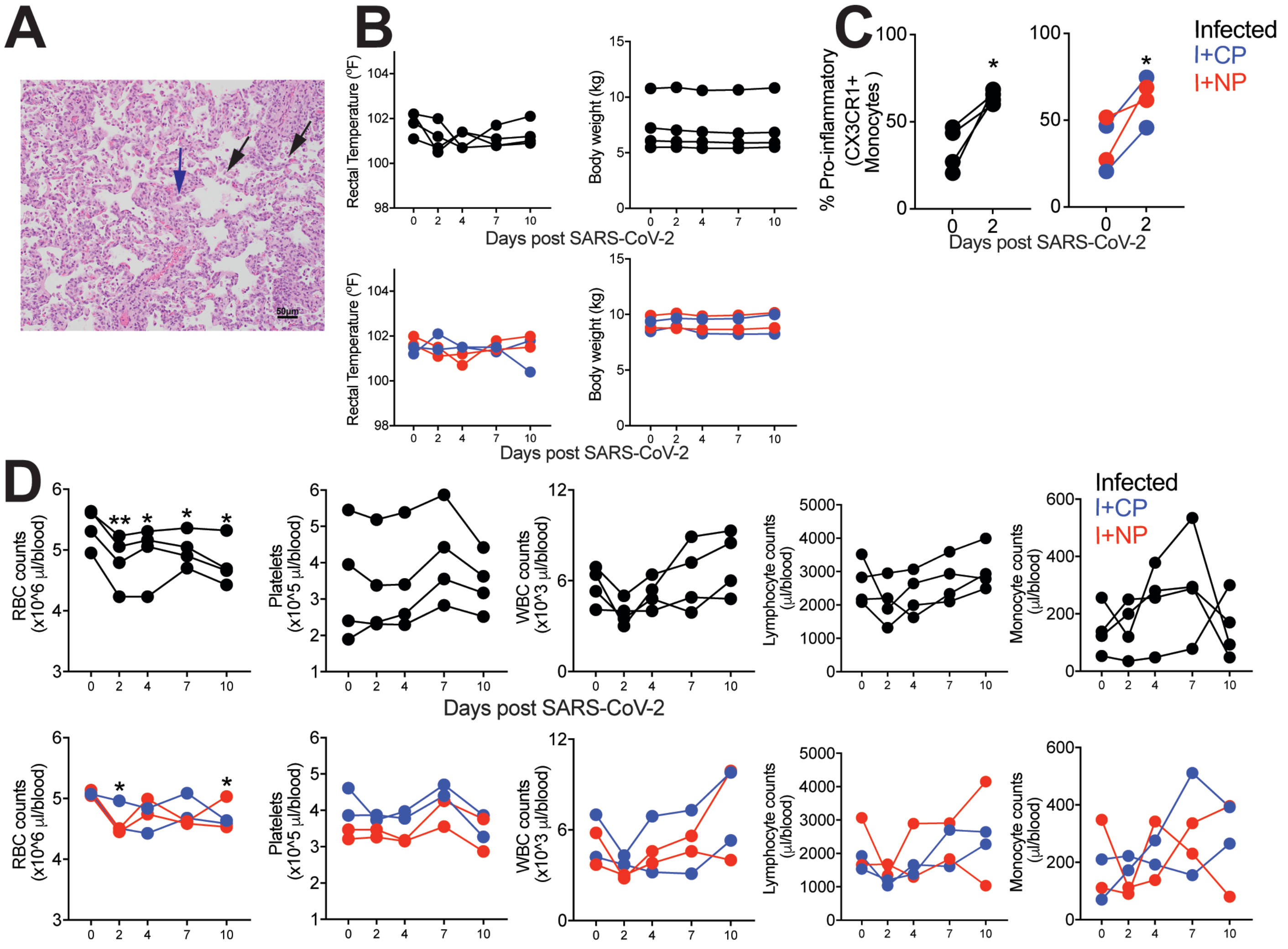

Supplemental Figure 1. Clinical symptoms and complete blood counts following SARS-CoV-2 infection (**A**) Alveolar septae are expanded by a mixed inflammatory cell infiltrate and alveoli contain macrophages [black arrow] and occasional neutrophils; interstitial thickening is also apparent [blue arrow]. H&E staining was performed in independent batches for all 8 animals and evaluated independently by two pathologists. Micrograph representative of animals in cohort (n=8). (**B**) Rectal Temperature [°F] and Body weight [kg] of SARS-CoV-2 infected rhesus macaques over the course of the study. (**C**) Frequency of pro-inflammatory monocytes [CD14+CD16+] expressing CX3CR1 at day 0 and 2 within blood following infection ($p = 0.02$ one-tailed paired t test for both infected and infected + infused animals. (**D**) RBC Counts [x10^6 /ul] (**, $p = 0.004$;* $p = 0.01$ at indicated time points relative to d0 using a one-tailed paired t test). Platelets [x10^5 /ul blood], WBC counts [x10^3 /ul blood], Lymphocyte counts [/ul blood] and Monocyte counts [/ul blood] over the course of the study; Infected (black circles) are RM that were infected and received no plasma treatment, I+CP (blue circles) are RM that were infected and received normal plasma from patients with no history of SARS-CoV-2 infection.

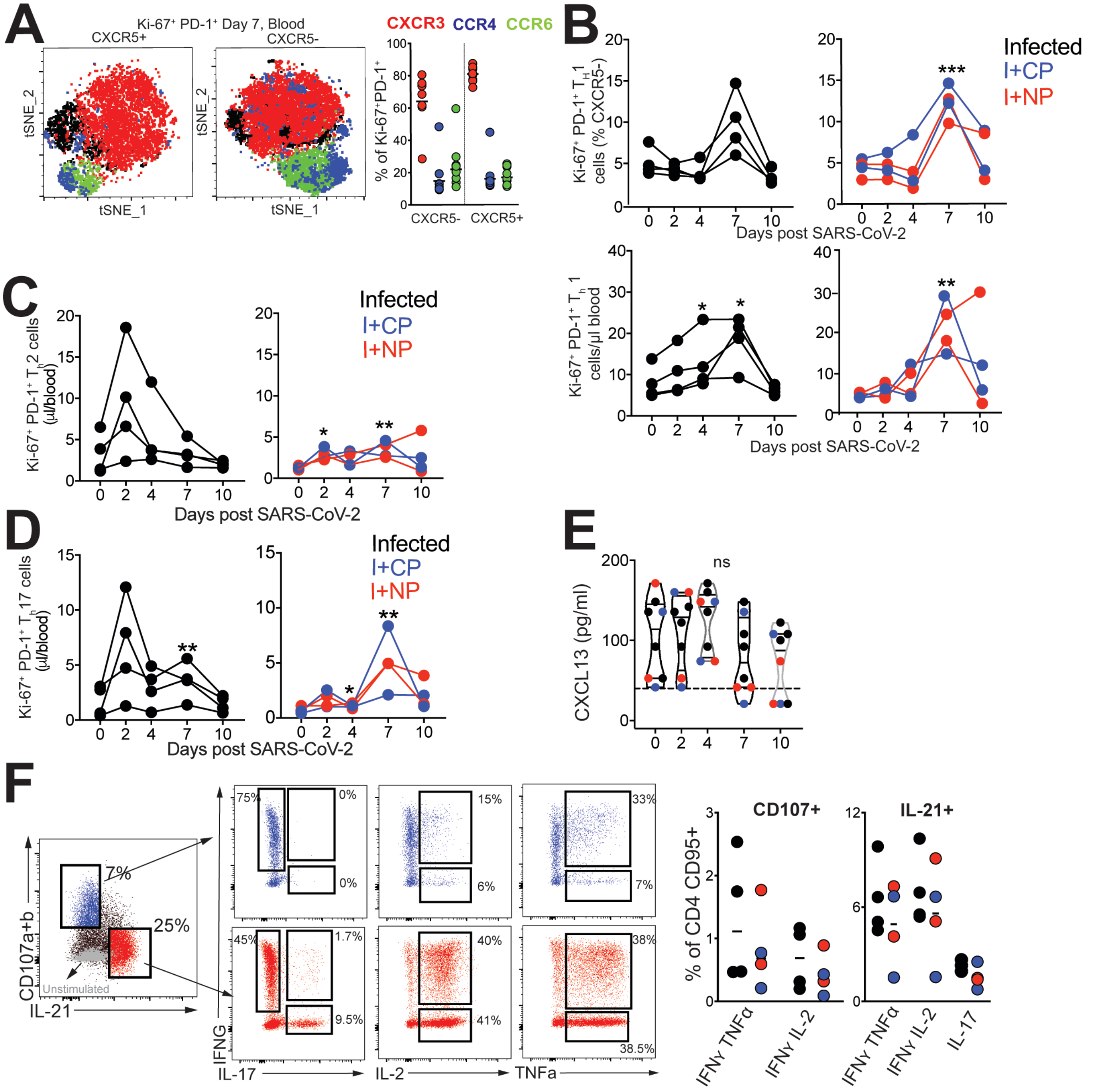

Supplemental Figure 2. SARS-CoV-2 infection elicits Ki67+PD-1+ Th1 cells at Day 7 that are polyfunctional (**A**) tSNE plots of CD4+Ki-67+PD-1+ CXCR5+/CXCR5- populations constructed from flow cytometry data show representation of Th1 effectors [CXCR3+], Th2 effectors [CCR4+], and Th17 effectors [CCR6+]. (**B**) Increase of Ki67+PD-1+ Th1 cells in both cell frequency and cells/ul blood at day 7 post infection. (**C**) Kinetics of Ki-67+ PD-1+ Th2 cells [/ul blood] (**D**) Kinetics of Ki-67+ PD-1+ Th17 cells [/ul blood]. (B-D, ***p = 0.001, **p = 0.0013, *p = 0.0153 at indicated time points relative to d0 using a one-tailed paired t test). (**E**) Serum CXCL13 following infection (ns using a one-tailed paired t test). Data points represent cytokine production from n = 8 animals. (**F**) Representative expression of IFNg, IL-17, IL-2, TNF-α within two CD4 populations: CD107a/b+ cells and IL-21+ cells after stimulation. Grey scatter plot in E shows overlay of cytokine production in unstimulated cells (unstim). Data points represent cytokine production from n = 8 animals.

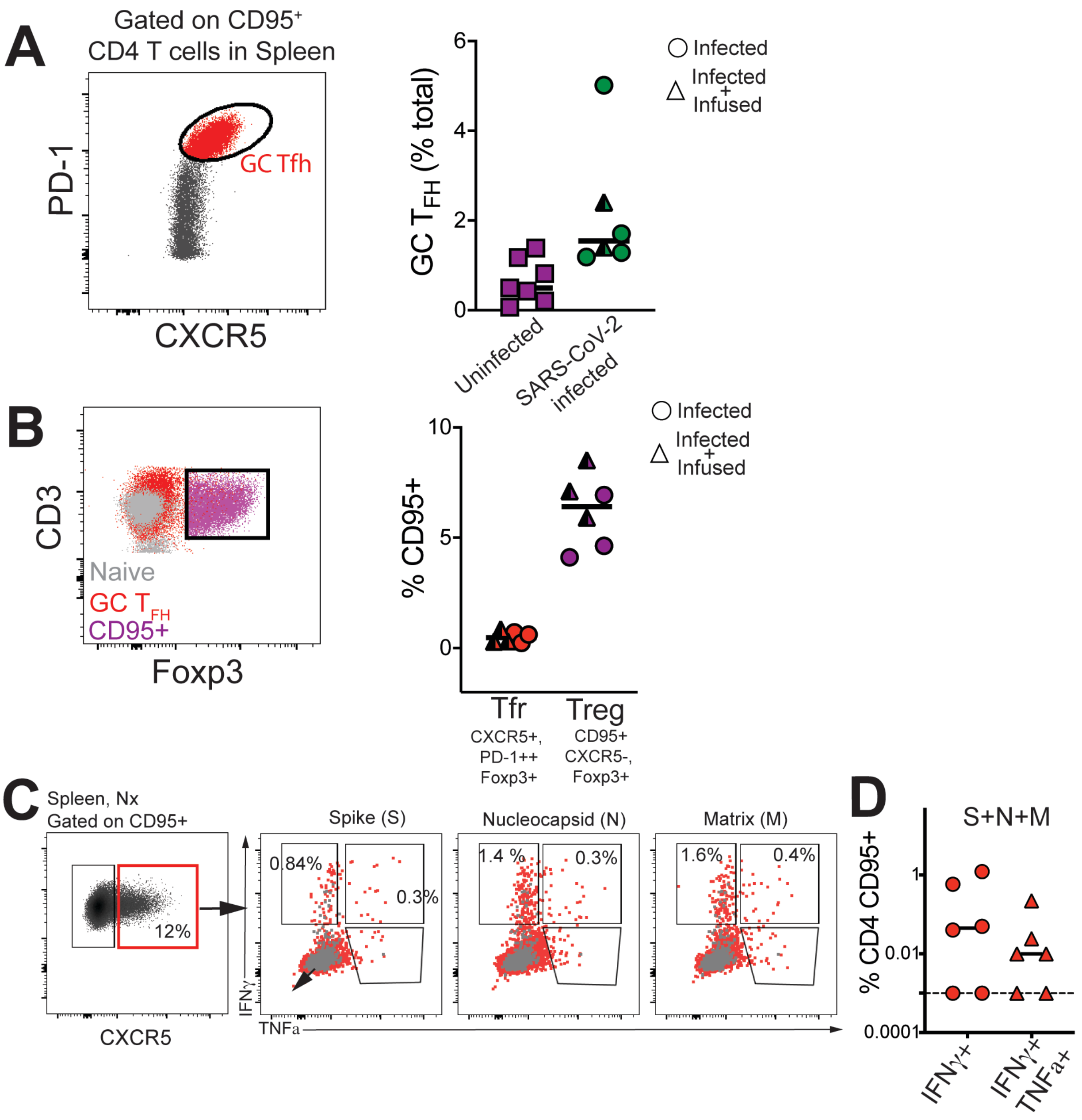

Supplemental Figure 3. Splenic GC TFH, Tfr, and Treg populations during SARS-CoV-2 infection (**A**) Flow plot of splenic GC TFH cells [CXCR5+PD-1hi] at necropsy (d11-d14pi) and dot plot graph designating an increase of GC TFH in SARS-CoV-2 infected rhesus macaques (n = 7 unexposed, n = 5 animals from SARS-CoV-2 infected animals). (**B**) Flow Plot of Treg [CD95+, CXCR5-, Foxp3+] and Tfr [CXCR5+,PD-1++, Foxp3+] cells. n = 5 animals from SARS-CoV-2 infected animals Controls (circles) are RM that were infected and received no plasma treatment, Infused (triangles) are RM that were infected and received either convalescent plasma or normal plasma. (**C**) Flow plot illustrating gating strategy to identify cytokine producing cells in spleen following stimulation with S, N, and M. Cytokine responses in unstimulated cells shown in gray. (**D**) Scatter plot shows frequency of cytokine+ CD4 T cells for n = 6 animals.

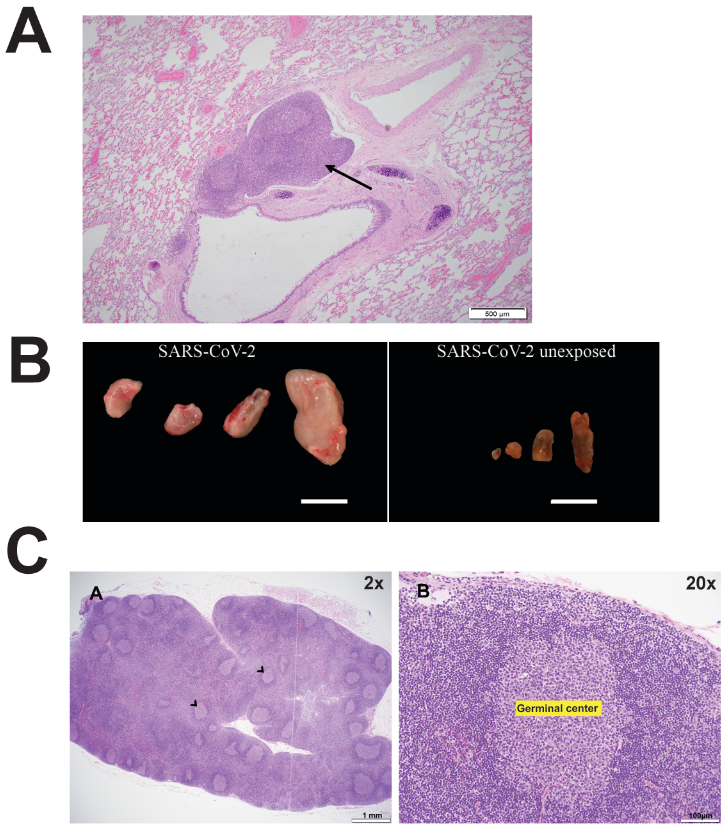

Supplemental Figure 4. SARS-CoV-2 infection increases mediastinal lymph node size and elicits germinal centers. (**A**) Large aggregate of bronchial associated lymphoid tissue [arrow] adjacent to a moderately large airway and associated blood vessel. Two well defined germinal centers can be seen within the BALT. H&E staining was performed in independent batches for all 8 animals and evaluated independently by two pathologists. Micrograph representative of animals in cohort (n=8). (**B**) Comparison of SARS-CoV-2 uninfected [left] and SARS-CoV-2 infected [right] mediastinal lymph nodes show distinct lymphadenopathy. (**C**) Representative H&E-stained mediastinal lymph node section showing distinct abundance of pale germinal centers (arrow). H&E staining was performed in a single batch and evaluated by a single technician. Image representative of GC within Mediastinal LN in all 8 animals.

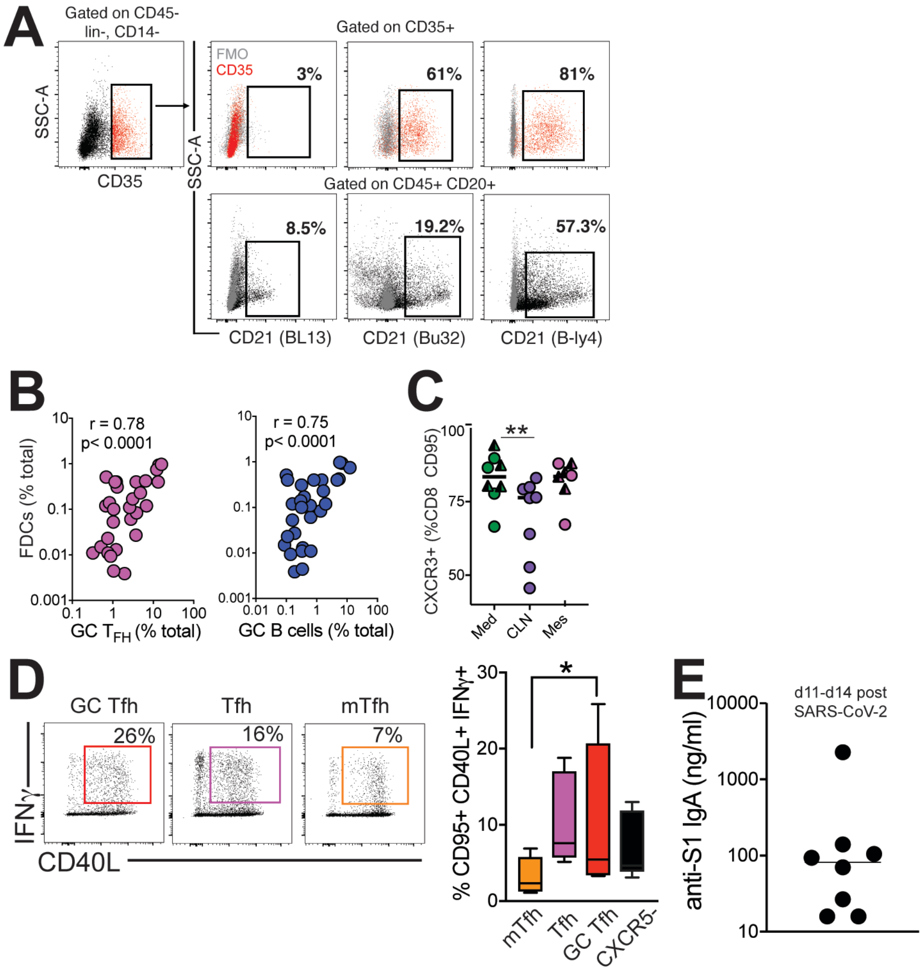

Supplemental Figure 5. SARS-CoV-2 infection elicits GC responses (**A**) Detection of follicular dendritic cells (FDCs) gating on CD45-Lin-CD14-CD35+CD21+ using flow cytometry; three different clones are used for comparison [BL13, BU32, B-LY4]. (**B**) Correlations between % total FDCs and % total GC TFH or % total GC B cells. Two-tailed Pearson test p values shown for 29 samples across lymph nodes from n = 8 animals. (**C**) Increased frequencies of CXCR3+ CD8 T cells in mediastinal lymph node (Med, n = 8; CLN, n = 8; Mes, n=7). (**D**) Functional characterization of mediastinal lymph node following SARS-CoV-2 infection in response to PMA/Ionomycin stimulation shows enrichment of CD40L+ IFNG+ cells within GC Tfh cells (* p< 0.05 relative to memory Tfh cells). Box-whiskers plot shows range of data, bounds of the box extend from the 25th to 75th percentile, line in box is plotted at the median. (**E**) IgA at necropsy
