# Supplementary material for: SARS-CoV-2 induces robust germinal center CD4 T follicular helper cell responses in rhesus macaques
Source: Nat Commun. 2021 Jan 22;12:541. doi: 10.1038/s41467-020-20642-x (PMC7822826; doi:10.1038/s41467-020-20642-x)
Supplement: Supplementary file 3 — Reporting Summary [file 41467_2020_20642_MOESM3_ESM.pdf]

nature research

Corresponding author(s): Smita Iyer

Last updated by author(s): Oct 22, 2020

Reporting Summary

Nature Research wishes to improve the reproducibility of the work that we publish. This form provides structure for consistency and transparency in reporting. For further information on Nature Research policies, see our Editorial Policies and the Editorial Policy Checklist.

Statistics

For all statistical analyses, confirm that the following items are present in the figure legend, table legend, main text, or Methods section.

n/a	Confirmed	
[ ]	[x]	The exact sample size (*n*) for each experimental group/condition, given as a discrete number and unit of measurement
[ ]	[x]	A statement on whether measurements were taken from distinct samples or whether the same sample was measured repeatedly
[ ]	[x]	The statistical test(s) used AND whether they are one- or two-sided<br>*Only common tests should be described solely by name; describe more complex techniques in the Methods section.*
[x]	[ ]	A description of all covariates tested
[x]	[ ]	A description of any assumptions or corrections, such as tests of normality and adjustment for multiple comparisons
[x]	[ ]	A full description of the statistical parameters including central tendency (e.g. means) or other basic estimates (e.g. regression coefficient) AND variation (e.g. standard deviation) or associated estimates of uncertainty (e.g. confidence intervals)
[x]	[ ]	For null hypothesis testing, the test statistic (e.g. *F*, *t*, *r*) with confidence intervals, effect sizes, degrees of freedom and *P* value noted<br>*Give P values as exact values whenever suitable.*
[x]	[ ]	For Bayesian analysis, information on the choice of priors and Markov chain Monte Carlo settings
[x]	[ ]	For hierarchical and complex designs, identification of the appropriate level for tests and full reporting of outcomes
[x]	[ ]	Estimates of effect sizes (e.g. Cohen's *d*, Pearson's *r*), indicating how they were calculated

Our web collection on statistics for biologists contains articles on many of the points above.

Software and code

Policy information about availability of computer code

Data collection	***1) Data Collection : Flow cytometry: FACSymphony (BD) with FACSDiva software version 8.0; ELISA: Bioplex 200 (BioRad); Serum cytokine: Luminex® 200 dual laser instrument (Luminex, Austin, TX); qPCR: QuantStudio 12K Flex Real-Time PCR System (Applied Biosystem)***
Data analysis	***2)Data analysis : Flow cytometry: FlowJo software version 10.; ELISA : BioManager software Serum cytokines analysis : xPONENT® software , Prism v9*** *software was used.*

For manuscripts utilizing custom algorithms or software that are central to the research but not yet described in published literature, software must be made available to editors and reviewers. We strongly encourage code deposition in a community repository (e.g. GitHub). See the Nature Research guidelines for submitting code & software for further information.

Data

Policy information about availability of data

All manuscripts must include a data availability statement. This statement should provide the following information, where applicable:

- Accession codes, unique identifiers, or web links for publicly available datasets
- A list of figures that have associated raw data
- A description of any restrictions on data availability

The data sets generated during and or analyses during the current study are available from the corresponding author on reasonable request.

Field-specific reporting

Please select the one below that is the best fit for your research. If you are not sure, read the appropriate sections before making your selection.

[x] Life sciences [ ] Behavioural & social sciences [ ] Ecological, evolutionary & environmental sciences

For a reference copy of the document with all sections, see nature.com/documents/nr-reporting-summary-flat.pdf

Life sciences study design

All studies must disclose on these points even when the disclosure is negative.

Sample size	We arrived at a sample size of 8 to capture acute and adaptive T cell responses based on the literature.
Data exclusions	No data were excluded from the analysis
Replication	We have subsequently capture immune kinetics and Tfh responses in independent SARS-CoV-2 studies and have observed similar kinetics in rhesus presenting in mild disease.
Randomization	Animals were randomized into experimental groups.
Blinding	The pathologists were blinded to the study design for the purpose of generating unbiased histopathological analyses.

Reporting for specific materials, systems and methods

We require information from authors about some types of materials, experimental systems and methods used in many studies. Here, indicate whether each material, system or method listed is relevant to your study. If you are not sure if a list item applies to your research, read the appropriate section before selecting a response.

Materials & experimental systems

n/a	Involved in the study	
[ ]	[x]	Antibodies
[x]	[ ]	Eukaryotic cell lines
[x]	[ ]	Palaeontology and archaeology
[ ]	[x]	Animals and other organisms
[x]	[ ]	Human research participants
[x]	[ ]	Clinical data
[x]	[ ]	Dual use research of concern

Methods

n/a	Involved in the study	
[x]	[ ]	ChIP-seq
[ ]	[x]	Flow cytometry
[x]	[ ]	MRI-based neuroimaging

Antibodies

Antibodies used

CD3-AF 700 SP34-2 BD Biosciences 557917; CD3-APC-Cy7 SP34-2 BD Biosciences 557757; CD4-BV 650 L200 BD Biosciences 563737
CD8-BV 510 SK-1 BD Biosciences 563919
CD20-APC-Cy7 2H7 BioLegend 302314
CD20-BV 421 2H7 BioLegend 302328
CD95-BUV 737 DX2 BD Biosciences 564710
CD279(PD-1)-Pe-Cy7 EH12.2H8 BioLegend 329918
CX3CR1- PE-CF594 2A9-1 BioLegend 341624
CXCR3-BV 786 IC6 BD Biosciences 741005
CXCR5-PE MU5UBEE Thermofischer 12-9185-411G1
CCR4-BV 605 1G1 BD Biosciences 562906
CCR6-PE-CF594/A610 G034E3 BioLegend 353430
CCR7-BV 650 3D12 BD Biosciences 563407
HLA-DR-BV 786 L243 BioLegend 307642
CD69-BV 711 FN50 BioLegend 310944
CD69-Pe-Cy7 FN50 Invitrogen 25-0699-42
CD14-AF 700 MSE2 BD Biosciences 301822
CD16-BV 605 3G8 BD Biosciences 563172
CD11b-BV 510 ICRF44 Thermofisher 563088
CD11c-Pe-Cy7 3.9 Invitrogen 25-0116-42
CD103-APC 2G5 Beckman Coulter B06204
CD66-APC TET2 Miltenyi 130-118-539
CD163-PE GHI/61 BioLegend 333606

CD123-BV 421 7G3 Thermofisher 501129764
Granzyme B-BV 421 GB11 BioLegend 515408
CD80-AF 488 2D10.4 Invitrogen 11-0809-42
CD86-AF 488 IT2.2 BioLegend 305414
Ki-67-AF 488 B56 BD Biosciences 558616
Ki-67-BV 510 B56 BD Biosciences 563462
CD28-Pe-Cy7 CD28.2 Tonbo 40-0289-U500
a4b7-PE Act-1 NHP Reag Res PR-1422
CD45-AF 488 D058-1283 BD Biosciences 557803
CD45-BV 605 D058-1283 BD Biosciences 564098
CD140b-APC 18A2 BioLegend 323608
Bcl-6-APC-Cy7 K112-91 BD Biosciences 563581
CD21- PE-CF594 B-ly4 BD Biosciences 563474
SLAM-AF 488 A12(7D4) BioLegend 306312
Foxp3-APC 206D BioLegend 320114
CD278 (ICOS)-BV 785 C396.4A BioLegend 313534
CD25-APC BC96 Tonbo 20-0259-T100
CD 134 (OX40)-BV 786 L106 BD Biosciences 744746
4-1BB-AF 488 4B4-1 BD Biosciences 11-1379-42
TNFa-AF 488 Mab11 BioLegend 502906
IL-21-APC 3A3-N2.1 BD Biosciences 560493
IFNG-PeCy7 B27 BioLegend 506518
IL2- PE-CF594 MO1-17H12 BioLegend 500344
CD107a-PE H4A3 BioLegend 328608
CD107b-PE EbioH4B4 Thermofischer 12-1078-42
IL-17-BV 421 eBio64DEC17 eBiosciences 48-7179-42
CD45 RO- PE-CF594 UCHL-1 BD Biosciences 562299
CD28-Purified Ab CD28.2 BD Biosciences 555725
CD49d-Purified Ab 9F10 BD Biosciences 555501
Live/dead-APC-Cy7 Life technologies L34976
Live/dead-BV 510 Life technologies L34966
Bcl-6 LN22 Biocare Medical CM410A
CD3 CD3-12 Abcam Ab11089
PD-1 Polyclonal Novus NBP1-88104

Validation

All antibodies were validated my the a manufacturer. In addition, our experiments included negative and positive controls.

Palaeontology and Archaeology

Specimen provenance

Provide provenance information for specimens and describe permits that were obtained for the work (including the name of the issuing authority, the date of issue, and any identifying information).

Specimen deposition

Indicate where the specimens have been deposited to permit free access by other researchers.

Dating methods

If new dates are provided, describe how they were obtained (e.g. collection, storage, sample pretreatment and measurement), where they were obtained (i.e. lab name), the calibration program and the protocol for quality assurance OR state that no new dates are provided.

☐ Tick this box to confirm that the raw and calibrated dates are available in the paper or in Supplementary Information.

Ethics oversight

Identify the organization(s) that approved or provided guidance on the study protocol, OR state that no ethical approval or guidance was required and explain why not.

Note that full information on the approval of the study protocol must also be provided in the manuscript.

Animals and other organisms

Policy information about studies involving animals; ARRIVE guidelines recommended for reporting animal research

Laboratory animals

Macaca mulatta, Males (n=5); Females (n=3); age 4 -5 years

Wild animals

The study did not involve wild animals

Field-collected samples

The study did not involve samples collected from the field

Ethics oversight

University of California at Davis

Note that full information on the approval of the study protocol must also be provided in the manuscript.

Human research participants

Policy information about studies involving human research participants

Population characteristics	*Describe the covariate-relevant population characteristics of the human research participants (e.g. age, gender, genotypic information, past and current diagnosis and treatment categories). If you filled out the behavioural & social sciences study design questions and have nothing to add here, write "See above."*
Recruitment	*Describe how participants were recruited. Outline any potential self-selection bias or other biases that may be present and how these are likely to impact results.*
Ethics oversight	*Identify the organization(s) that approved the study protocol.*

Note that full information on the approval of the study protocol must also be provided in the manuscript.

Clinical data

Policy information about clinical studies

All manuscripts should comply with the ICMJE guidelines for publication of clinical research and a completed CONSORT checklist must be included with all submissions.

Clinical trial registration	*Provide the trial registration number from ClinicalTrials.gov or an equivalent agency.*
Study protocol	*Note where the full trial protocol can be accessed OR if not available, explain why.*
Data collection	*Describe the settings and locales of data collection, noting the time periods of recruitment and data collection.*
Outcomes	*Describe how you pre-defined primary and secondary outcome measures and how you assessed these measures.*

Dual use research of concern

Policy information about dual use research of concern

Hazards

Could the accidental, deliberate or reckless misuse of agents or technologies generated in the work, or the application of information presented in the manuscript, pose a threat to:

No	Yes	
☒	☐	Public health
☒	☐	National security
☒	☐	Crops and/or livestock
☒	☐	Ecosystems
☒	☐	Any other significant area

Experiments of concern

Does the work involve any of these experiments of concern:

No	Yes	
☒	☐	Demonstrate how to render a vaccine ineffective
☒	☐	Confer resistance to therapeutically useful antibiotics or antiviral agents
☒	☐	Enhance the virulence of a pathogen or render a nonpathogen virulent
☒	☐	Increase transmissibility of a pathogen
☒	☐	Alter the host range of a pathogen
☒	☐	Enable evasion of diagnostic/detection modalities
☒	☐	Enable the weaponization of a biological agent or toxin
☒	☐	Any other potentially harmful combination of experiments and agents

ChIP-seq

Data deposition

☐ Confirm that both raw and final processed data have been deposited in a public database such as GEO.

☐ Confirm that you have deposited or provided access to graph files (e.g. BED files) for the called peaks.

Data access links
May remain private before publication.

For "Initial submission" or "Revised version" documents, provide reviewer access links. For your "Final submission" document, provide a link to the deposited data.

Files in database submission

Provide a list of all files available in the database submission.

Genome browser session
(e.g. UCSC)

Provide a link to an anonymized genome browser session for "Initial submission" and "Revised version" documents only, to enable peer review. Write "no longer applicable" for "Final submission" documents.

Methodology

Replicates

Describe the experimental replicates, specifying number, type and replicate agreement.

Sequencing depth

Describe the sequencing depth for each experiment, providing the total number of reads, uniquely mapped reads, length of reads and whether they were paired- or single-end.

Antibodies

Describe the antibodies used for the ChIP-seq experiments; as applicable, provide supplier name, catalog number, clone name, and lot number.

Peak calling parameters

Specify the command line program and parameters used for read mapping and peak calling, including the ChIP, control and index files used.

Data quality

Describe the methods used to ensure data quality in full detail, including how many peaks are at FDR 5% and above 5-fold enrichment.

Software

Describe the software used to collect and analyze the ChIP-seq data. For custom code that has been deposited into a community repository, provide accession details.

Flow Cyto[illegible]

We have provided marker and fluorochrome used in the Figure legends

Plots

Confirm that:

- [ ] The axis labels state the marker and fluorochrome used (e.g. CD4-FITC).
- [x] The axis scales are clearly visible. Include numbers along axes only for bottom left plot of group (a 'group' is an analysis of identical markers).
- [x] All plots are contour plots with outliers or pseudocolor plots.
- [x] A numerical value for number of cells or percentage (with statistics) is provided.

Methodology

Sample preparation

Whole blood was use fresh, Lung, spleen, and lymph node samples were digested with collagenase to isolate single cell suspensions.

Instrument

BD FACS Symphony

Software

FlowJo v10

Cell population abundance

No sorting was performed

Gating strategy

Representative gating strategies for all populations are provided in the manuscript

- [x] Tick this box to confirm that a figure exemplifying the gating strategy is provided in the Supplementary Information.

Magnetic resonance imaging

Experimental design

Design type

Indicate task or resting state; event-related or block design.

Design specifications

Specify the number of blocks, trials or experimental units per session and/or subject, and specify the length of each trial or block (if trials are blocked) and interval between trials.

Behavioral performance measures

State number and/or type of variables recorded (e.g. correct button press, response time) and what statistics were used to establish that the subjects were performing the task as expected (e.g. mean, range, and/or standard deviation across subjects).

Acquisition

Imaging type(s) | *Specify: functional, structural, diffusion, perfusion.*

Field strength | *Specify in Tesla*

Sequence & imaging parameters | *Specify the pulse sequence type (gradient echo, spin echo, etc.), imaging type (EPI, spiral, etc.), field of view, matrix size, slice thickness, orientation and TE/TR/flip angle.*

Area of acquisition | *State whether a whole brain scan was used OR define the area of acquisition, describing how the region was determined.*

Diffusion MRI ☐ Used ☐ Not used

Preprocessing

Preprocessing software | *Provide detail on software version and revision number and on specific parameters (model/functions, brain extraction, segmentation, smoothing kernel size, etc.).*

Normalization | *If data were normalized/standardized, describe the approach(es): specify linear or non-linear and define image types used for transformation OR indicate that data were not normalized and explain rationale for lack of normalization.*

Normalization template | *Describe the template used for normalization/transformation, specifying subject space or group standardized space (e.g. original Talairach, MNI305, ICBM152) OR indicate that the data were not normalized.*

Noise and artifact removal | *Describe your procedure(s) for artifact and structured noise removal, specifying motion parameters, tissue signals and physiological signals (heart rate, respiration).*

Volume censoring | *Define your software and/or method and criteria for volume censoring, and state the extent of such censoring.*

Statistical modeling & inference

Model type and settings | *Specify type (mass univariate, multivariate, RSA, predictive, etc.) and describe essential details of the model at the first and second levels (e.g. fixed, random or mixed effects; drift or auto-correlation).*

Effect(s) tested | *Define precise effect in terms of the task or stimulus conditions instead of psychological concepts and indicate whether ANOVA or factorial designs were used.*

Specify type of analysis: ☐ Whole brain ☐ ROI-based ☐ Both

Statistic type for inference (See Eklund et al. 2016) | *Specify voxel-wise or cluster-wise and report all relevant parameters for cluster-wise methods.*

Correction | *Describe the type of correction and how it is obtained for multiple comparisons (e.g. FWE, FDR, permutation or Monte Carlo).*

Models & analysis

n/a	Involved in the study
☐	☐ Functional and/or effective connectivity
☐	☐ Graph analysis
☐	☐ Multivariate modeling or predictive analysis

Functional and/or effective connectivity | *Report the measures of dependence used and the model details (e.g. Pearson correlation, partial correlation, mutual information).*

Graph analysis | *Report the dependent variable and connectivity measure, specifying weighted graph or binarized graph, subject- or group-level, and the global and/or node summaries used (e.g. clustering coefficient, efficiency, etc.).*

Multivariate modeling and predictive analysis | *Specify independent variables, features extraction and dimension reduction, model, training and evaluation metrics.*
